# Visual processing of multiple elements in the dyslexic brain: evidence for a superior parietal dysfunction

**DOI:** 10.3389/fnhum.2014.00479

**Published:** 2014-07-07

**Authors:** Muriel A. Lobier, Carole Peyrin, Cédric Pichat, Jean-François Le Bas, Sylviane Valdois

**Affiliations:** ^1^Laboratoire de Psychologie et NeuroCognition, Université Grenoble AlpesGrenoble, France; ^2^Neuroscience Center, University of HelsinkiHelsinki, Finland; ^3^CNRS, Laboratoire de Psychologie et NeuroCognitionUMR5105, Grenoble, France; ^4^INSERM U836/Université Joseph Fourier – Institut des NeurosciencesGrenoble, France

**Keywords:** developmental dyslexia, visual attention, reading, superior parietal lobes

## Abstract

The visual attention (VA) span deficit hypothesis of developmental dyslexia posits that impaired multiple element processing can be responsible for poor reading outcomes. In VA span impaired dyslexic children, poor performance on letter report tasks is associated with reduced parietal activations for multiple letter processing. While this hints towards a non-specific, attention-based dysfunction, it is still unclear whether reduced parietal activity generalizes to other types of stimuli. Furthermore, putative links between reduced parietal activity and reduced ventral occipito-temporal (vOT) in dyslexia have yet to be explored. Using functional magnetic resonance imaging, we measured brain activity in 12 VA span impaired dyslexic adults and 12 adult skilled readers while they carried out a categorization task on single or multiple alphanumeric or non-alphanumeric characters. While healthy readers activated parietal areas more strongly for multiple than single element processing (right-sided for alphanumeric and bilateral for non-alphanumeric), similar stronger multiple element right parietal activations were absent for dyslexic participants. Contrasts between skilled and dyslexic readers revealed significantly reduced right superior parietal lobule (SPL) activity for dyslexic readers regardless of stimuli type. Using a priori anatomically defined regions of interest, we showed that neural activity was reduced for dyslexic participants in both SPL and vOT bilaterally. Finally, we used multiple regressions to test whether SPL activity was related to vOT activity in each group. In the left hemisphere, SPL activity covaried with vOT activity for both normal and dyslexic readers. In contrast, in the right hemisphere, SPL activity covaried with vOT activity only for dyslexic readers. These results bring critical support to the VA interpretation of the VA Span deficit. In addition, they offer a new insight on how deficits in automatic vOT based word recognition could arise in developmental dyslexia.

## INTRODUCTION

Developmental dyslexia is a severe, persistent reading disability: dyslexic children and adults do not acquire efficient, fluent reading despite adequate schooling and intelligence. A large body of research has supported difficulties with language processing (Bishop and Snowling, 2004) and more specifically with phonological processing of oral language as the core deficit in dyslexia (Ramus, 2003; Vellutino et al., 2004; Ramus and Szenkovits, 2008). Accordingly, numerous studies have reported links between phonological deficits and left hemisphere language areas neural dysfunction in developmental dyslexia (see Démonet et al., 2004; Maisog et al., 2008; Richlan et al., 2009, 2011 for reviews). In addition, developmental dyslexia has been associated with disrupted activity in the left ventral occipito-temporal (vOT) cortex (Richlan et al., 2009, 2011; Van der mark et al., 2011) thought to subserve visual processing of letter strings (Dehaene and Cohen, 2011). However, in accordance with multifactorial accounts of dyslexia (Pennington, 2006; Menghini et al., 2010; Vidyasagar and Pammer, 2010), recent research has hinted towards a possible visual component to the core deficit in dyslexia. Various deficits in visual attention (VA) and visual processing have been identified in dyslexic individuals as supporting different visual-attentional models of developmental dyslexia (Hari and Renvall, 2001; Facoetti et al., 2006, 2008; Boden and Giaschi, 2007; Bosse et al., 2007; Vidyasagar and Pammer, 2010). Most of these models assume the co-occurrence of VA and phonological deficits in dyslexic individuals except the VA span model which posits that a deficit in multi-element (ME) visual processing can account for reading acquisition problems in a subset of dyslexic individuals who otherwise have preserved phonological skills (Valdois et al., 2004, 2014b; Bosse et al., 2007).

Indeed, according to both case studies (Valdois et al., 2003; Dubois et al., 2010) and group studies (Bosse et al., 2007; Lassus-Sangosse et al., 2008), a subset of dyslexic children suffers from a selective deficit in multiple letter report tasks, independently from any phonological deficit. Performance on report tasks is interpreted as indexing the number of individual elements that can be processed in parallel, i.e., the VA Span. Impaired performance is thus viewed as a consequence of a reduced VA Span: dyslexic children cannot process as many letters in parallel as normal reading children. Furthermore, within the theoretical framework of the MultiTrace Memory (MTM) model (Ans et al., 1998), a reduced VA Span also results in impaired reading performance. According to the MTM model, letters of a word are processed in parallel through a visual-attention window. In expert readers, the size of this window adapts to the length of the to-be-read word in order to encompass all of its letter string. If the to-be-read word is unfamiliar, the window’s size is subsequently reduced to cover fewer letters and focus on the word orthographic units (letters, graphemes, or syllables). Reading then switches from a fast, parallel procedure to a slow, serial identification of successive orthographic units. If a deficit in visual processing capacity limits the ability of the visual-attention window to spread over a whole word, then words cannot be identified by a fast, parallel procedure resulting in impaired reading ability (for a more detailed and complete theoretical overview of the role of VA Span in impaired reading, see Valdois et al., 2004).

The VA Span definition places no constraints on the visual elements to which it refers: they may be letters or other visual elements. In turn, the VA Span deficit hypothesis posits that the ME processing deficit it evidences extends to any type of visual element, independently of its lexical nature. However, it has been suggested that low performance in letter report tasks using both verbal report and verbal stimuli (letters or digits) follows not from a deficit in visual processing but from impaired mapping of visual codes onto phonology (Hawelka and Wimmer, 2008; Ziegler et al., 2010). This hypothesis is supported by data suggesting that normal readers’ performance on a two alternative forced choice partial report task is higher than dyslexic readers’ for letters and digits but not symbols (Ziegler et al., 2010). However, other studies have brought forward evidence for a ME deficit that extends to non-verbal tasks and stimuli. Dyslexic adults and children are impaired on a symbol-string matching task requiring no verbal report (Pammer et al., 2004; Jones et al., 2008). A recent study used a non-verbal ME visual processing task to explore visual processing performance on non-verbal character strings in dyslexic children chosen to have a VA span disorder (Lobier et al., 2012b). In this task, a five element string made up of characters belonging to two different categories (e.g., pseudo-letters/unknown geometrical shapes, letters/digits) was displayed for 200 ms and then masked. Participants were asked to identify how many characters in the displayed string belonged to a previously designated target category. VA span impaired dyslexic children showed lower performance than age-matched controls, regardless of target character category. Since this categorization task required no verbal response and since no visual to phonological code mappings exist for novel target characters, these results argue strongly for an underlying visual processing impairment in the VA Span deficit (see Valdois et al., 2012, for converging evidence against the visual to phonological code mapping hypothesis). The prevalence of the VA Span deficit in the dyslexic population has been previously estimated in cohorts of dyslexic children. Around a third of dyslexic children were found to exhibit an isolated VA Span deficit in either French (Bosse et al., 2007; Zoubrinetzky et al., 2014), British (Bosse et al., 2007), or Brazilian Portuguese (Germano et al., submitted).

Abnormal neural activity in brain areas associated with VA in VA Span impaired children has brought forward additional evidence for VA as a constraining factor of VA Span performance in dyslexia. Neural correlates of the VA Span deficit were first explored in an functional magnetic resonance imaging (fMRI) study comparing neural activity for a flanked letter categorization task between normal reading and VA Span impaired dyslexic children (Peyrin et al., 2011). VA mechanisms involved in multi-letter processing were assessed using a task that minimized verbal report and phonological processing. Results showed that superior parietal lobule (SPL) activity was reduced bilaterally in dyslexic children compared to controls. Importantly, a recent case report (Peyrin et al., 2012) suggested that this SPL dysfunction is specific to the VA span deficit rather than to dyslexia. Neural activity for the same visual categorization task was assessed in two dyslexic adults with distinct neurocognitive profiles. SPL activity was normal for the patient with a phonological deficit but preserved VA span performance whereas it was decreased for the patient with a VA span deficit but preserved phonological performance.

The co-occurrence of poor multiple letter report performance and SPL dysfunction is consistent with a visuo-attentional account of the VA span disorder. SPL activity has not only been associated with visuo-spatial attention (Wojciulik and Kanwisher, 1999; Corbetta and Shulman, 2002; Behrmann et al., 2004) but also, more specifically, with ME processing (Mitchell and Cusack, 2008; Xu and Chun, 2009; Scalf and Beck, 2010). Closer to the cognitive demands of reading, SPL activity relates to length effects in pseudo-word reading (Valdois et al., 2006) and is observed in proficient readers when word letter parallel identification is compromised (Cohen et al., 2008 see also Gaillard et al., 2006). If SPL plays a role in reading acquisition, it should show different patterns of activation for different levels of reading proficiency. Indeed, less proficient readers have stronger bilateral (children vs. adults, see Church et al., 2008), right lateralized (Adult ex-illiterates vs. literates, see Dehaene et al., 2010) posterior parietal activity than more proficient readers. In addition, activity in left SPL and right IPL/SPL clusters is negatively correlated with reading proficiency (Jobard et al., 2011). In line with this putative role of SPL in reading acquisition, Brem et al. (2010) report activity peaks in right SPL for visual word processing in learning to read children. In Chinese, Cao et al. (2010) shows developmental increases in bilateral SPL during visuo-orthographic processing and stronger involvement of the right SPL during the visual comparison of two-character words than during phonological processing of the same words.

We recently showed stronger SPL involvement for pre-orthographic processing of multiple character strings than of single flanked characters, for both alphanumeric (AN) and non-alphanumeric (nAN) characters (Lobier et al., 2012a). However, this reduced SPL activity has only been reported for multiple letter processing, which cannot disentangle between a general ME impairment or a more specific letter processing impairment. A stronger argument for a VA dysfunction as the underlying factor in VA Span impairment would be made by showing a similar SPL dysfunction in dyslexic participants on a non-verbal ME task using both verbal and non-verbal stimuli.

The main aim of this study is to use non-verbal categorization tasks to isolate the underlying neural dysfunction in the VA Span disorder in dyslexia using fMRI. VA span impaired dyslexic adults and healthy skilled adult readers carried out a visual categorization with either alphanumeric, familiar characters or non-alphanumeric, unfamiliar characters. In order to isolate neural correlates specific to parallel processing of MEs, the task had two conditions: a ME categorization condition of interest and a single-element (SE) categorization control condition. Both conditions were carried out with either AN or nAN characters. While both the experimental and control conditions required visual categorization of the attended stimuli, only the experimental condition required processing of several elements. Contrasts between these conditions should highlight neural activations that are specific to ME processing demands.

Our central hypothesis is that the VA span deficit is associated with disrupted SPL activity for pre-orthographic multiple character processing regardless of character type. In line with previous studies, we expect to find abnormal parietal activations for multiple-element processing for the dyslexic group. More importantly, these abnormal brain activations should be found regardless of stimuli type. We first contrasted whole-brain neural activity between VA span impaired dyslexic adults and control normal-reading adults. In addition, we used regions of interest (ROIs) to compare more specifically activity in inferior parietal and superior parietal cortices between groups. Finally, since abnormal activity in the vOT cortex is commonly reported for dyslexic readers, we also used ROIs to test whether SPL activity was correlated with vOT activity.

## MATERIALS AND METHODS

### PARTICIPANTS

Twelve dyslexic (mean age 21.6 ± 4.2 years) and twelve healthy, skilled adult readers (mean age 23.8 ± 2.6 years) took part in this study. They were all right-handed and had normal or corrected to normal vision. All participants had given informed consent and received 60 Euros for their participation. Dyslexic participants were recruited through the university disabilities office. They had previously undergone a complete neuropsychological assessment to establish the diagnosis of developmental dyslexia and the presence of a VA span disorder while ruling out any co-morbid attentional disorders (e.g., ADHD). The diagnosis of developmental dyslexia was established using both inventories and testing procedures in accordance with the guidelines of the ICD-10 classification of Mental and Behavioral disorders. Reading speed was estimated for all participants, using the “Alouette” text (Lefavrais, 1965) that required reading a 265 word text as quickly and as accurately as possible during 3 min. Control participants had no reported learning or reading disability. Reading speed for dyslexic participants was significantly lower than for control participants (Dyslexic: Mean = 119wpm, 95%CI = [103–135], Controls: Mean = 202wpm, 95%CI = [185–219], *t*(22) = 7.9, *p* < 0.0001). This study was approved by the local ethics committee.

### VISUAL ATTENTION SPAN ASSESSMENT

All participants carried out a global letter report task in order to assess their VA span abilities. Ceiling effects are often observed for adults on the 5-letter report task used in previous studies with children (Valdois et al., 2003; Bosse et al., 2007). For this reason, a 6-letter report task was developed for testing adults (Peyrin et al., 2012). Stimuli were random 6-consonant strings presented in black upper-case letters on a white background. At the start of each trial, a central fixation point was displayed for 1000 ms followed by a 50 ms blank screen. A horizontal 6-letter string was then presented for 200 ms, centered on fixation. Participants were asked to report all the letters they had seen with no time pressure. Ten training and 24 experimental trials were carried out. Experimental stimuli were 24 consonant strings built-up from 10 consonants (BPTFLMDSRH). An additional 10 different letter strings were used for training. Score was the number of accurately reported letters, regardless of order (maximum score: 144).

The VA span performance of the participants was compared to normative data from the EVADYS diagnostic tool (Valdois et al., 2014a). Every control participant scored within 1 SD of the norm on the VA span task. The dyslexic participants’ VA Span abilities were at least 1.65 standard deviations below adult norms. Performance on the 6-letter whole report task indexing ME processing capacity (VA Span) was significantly lower for dyslexic (3.5 letters per trial on average) than for control (5.3 letters) participants (Dyslexic: Mean score = 84, 95%CI = [74–94], Control: Mean score = 128, 95%CI = [123–133], *t*(16.4) = 9.0, *p* < 0.0001).

### fMRI STUDY

#### Stimuli

Four different character categories were used: letters, digits, Japanese Hiragana, and pseudo-letters, with five different characters in each category. While participants had extensive multiple character processing experience with two categories (letters and digits), the other two were completely novel. The font used for letters and digits was Arial. Letters were drawn from the following set of five consonants: D, F, K, M, and V. Digits were drawn from the following set of five digits 3, 5, 6, 8, and 9. Pseudoletters were taken from a set created by Hawelka and Wimmer (2008) by cutting and rearranging letter visual features. The five characters created from consonants D, F, K, M, and V made up the pseudo-letter set. The five Hiragana characters were chosen amongst the 48 possible characters of the Hiragana syllabary so that their mean visual complexity as defined by Majaj et al. (2002), was similar to that of the other character sets. Character perimetric complexity is a reliable predictor of character recognition efficiency (Pelli et al., 2006): characters sets with similar average perimetric complexity are recognized with similar efficiency.

For the ME condition, strings of five characters were built-up from these sets. There were 48 AN strings and 48 nAN strings. Out of the 48 AN strings, 24 were consistent and 24 were inconsistent. Consistent strings were made up exclusively of letters and digits. Twelve of the consistent strings contained three letters and two digits and the other 12 contained two letters and three digits. Inconsistent strings were made up of letters, digits and one distractor character, either Hiragana or pseudo-letter. Twelve of the inconsistent strings contained two letters, two digits and one distractor character and the other 12 contained three letters, one digit and one distractor character. The position and choice of the distractor character was controlled across trials. Similarly, individual character positions were counterbalanced across consistent and inconsistent trials. The 48 nAN strings were built up the same way as the AN ones, with pseudo-letters and Hiragana replacing letters and digits. Distractor characters were then letters and digits. For the SE condition, stimuli were made up of one central character surrounded by four pound (#) signs. There were 48 strings: 24 with a central AN character (12 letters, 12 digits) and 24 with a central nAN character (12 pseudo-letters, 12 Hiragana). For all stimulus strings, characters subtended a visual angle of 0.7°. To minimize visual crowding, the distance between adjacent characters was of 0.57°. The entire string subtended a visual angle of 5.4° and was drawn in white on a black background.

#### Procedure

**FIGURE 1:**
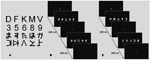
**Character sets and fMRI task procedure. (A)** Character sets (letters, digits, Hiragana, pseudo-letters). **(B)** Procedure screens for the single element task. **(C)** Procedure screens for the multiple element task.

A task requiring visual categorization of characters was carried out in two conditions: ME and SE (see **Figure 1**). Stimuli were displayed for 200 ms to avoid useful ocular saccades and serial visual processing. Stimuli display was driven by E-Prime software (E-Prime Psychology Software Tools, Inc., Pittsburgh, USA). Synchronization between scanner and paradigm was ensured by a trigger pulse sent from the scanner to the computer on which E-Prime was running. The paradigm was presented using a video projector (Epson EMP 8200), a projection screen situated behind the magnet and a surface mirror centered above the participant’s eyes. A response key was used to collect participant responses. Response accuracy and reaction times (RT, in milliseconds) were recorded.

In the ME condition, visual categorization of individual characters of a ME string was required. Performance was monitored by asking participants to report the number of target category characters present in the stimulus string. For AN strings, participants were asked to report the number of letters present in a letter and digit 5-character string. For nAN strings, participants were asked to report the number of Hiragana characters in a Hiragana and pseudo-letter character string. Participants pressed the index finger button for two target-category characters and the middle finger button for three target-category characters. They carried out 48 trials for each condition, half with two target characters and half with three target characters. Trial order was pseudo-randomized.

In the SE condition, visual categorization of a single character flanked by pound signs was required. Performance was monitored by asking participants to report whether or not the stimulus character belonged to either one of two target categories (AN: letters or digits, nAN: Hiragana or pseudo-letters). If the stimulus character belonged to a target category, participants pressed the index finger button. If it did not, they pressed the middle finger button. They carried out 48 trials for each condition, half of which contained a target category character. Trial order was pseudo-randomized. This condition was designed to control for three important task characteristics. First, low-level visual stimulation was similar to the ME condition: five characters were displayed (four pound signs and a central stimulus character). Second, motor response was the same for both tasks. Last, both conditions required character categorization, controlling for higher-order categorization processing.

Immediately before the scanning session, participants took part in a 45 min training session. Participants first performed two character-identification tasks in order to familiarize themselves with the two unfamiliar character types. During the second part of training, participants were familiarized with the experimental task. For each condition (ME and SE) and each character type (AN and nAN), they first carried out five training trials followed by a sequence of 48 trials with the same timing as the experimental sequence (but different stimulus strings).

### EVENT-RELATED fMRI EXPERIMENTAL DESIGN

Each participant carried out four event-related-fMRI sessions: two to assess ME processing (one for AN and one for nAN characters) and the other two to assess SE processing (one for AN and one for nAN characters). FMRI session order was counterbalanced across participants. Stimuli onsets were optimized using pseudo-randomized ER-fMRI paradigms (Friston et al., 1999). For each session, 48 stimulus strings were displayed: 24 consistent and 24 inconsistent. In order to provide an appropriate baseline measure (Friston et al., 1998), 27 null-events (three of them at the end of the session) were included in each session. These null-events comprised a black screen and a fixation dot displayed at the center of the screen. SOA between events was set to 3 s. SOAs between trial events were of 3, 6, or 9 s, depending on the presence of null-events. To reduce eye movements, participants were asked to fixate the fixation dot during null-events. In order to stabilize the magnetic field, each functional run started with five dummy scans that were discarded before analysis. After these dummy scans, 90 functional volumes were acquired for each run. Each functional session lasted 3 min 45 s.

### MR ACQUISITION

A whole-body 3T MR scanner was used (Bruker MedSpec S300) with 41 mT/m maximum gradient strength and 120 mT/m/s maximum slew rate. For functional scans, the manufacturer-provided gradient-echi/T2^*^ weighted EPI method was used. Thirty-nine adjacent axial slices parallel to the bi-commissural plane were acquired in interleaved mode. Slice thickness was 3.5 mm. The in-plane voxel size was 3 mm × 3 mm (216 × 216 field of view acquired with a 72 × 72 pixels data matrix; reconstructed with 0 filling to 128 × 128 pixels). The main sequence parameters were: TR = 2.5 s, TE = 30 ms, flip angle = 80°. To correct images for geometric distortions induced by local B0- inhomogeneity, a B0 fieldmap was derived from two gradient echo data sets acquired with a standard 3D FLASH sequence ΔTE = 9.104 ms). The fieldmap was subsequently used during data processing. Finally, a T1-weighted high-resolution three dimensional anatomical volume was acquired, by using a sagittal magnetization-prepared rapid acquisition gradient echo (MP-RAGE) sequence (field of view = 256 × 224 × 176 mm; resolution = 1.333 × 1.750 × 1.375 mm; acquisition matrix: 192 × 128 × 128 pixels; reconstruction matrix = 256 × 128 × 128 pixels).

### DATA PROCESSING

Both preprocessing and statistical analyses of the data were performed using the Statistical Parametric Mapping software (SPM5, Wellcome Department of Imaging Neuroscience, London, UK; http://www.fil.ion.ucl.ac.uk/spm; Friston et al. (1994). Functional volumes were time corrected using the 20th slice as reference. All volumes were then realigned using rigid body transformations to correct for head movement, using the first ER-fMRI session as the reference volume. The T1-weighted anatomical volume was co-registered to the realigned mean images and normalized to MNI space using a trilinear interpolation. The anatomical normalization parameters were then used for functional volume normalization. Finally, each functional volume was smoothed by an 8-mm FWHM (Full Width at Half Maximum) Gaussian kernel. Time series for each voxel were high-pass filtered (1/128 cut-off) to remove low-frequency noise and signal drift.

### STATISTICAL ANALYSES

#### Whole-brain analyses

Statistical analyses were performed on the pre-processed functional images for each one of the four sessions. For each session (ME AN and nAN, SE AN and nAN), consistency (consistent and inconsistent character strings) was modeled as a regressor convolved with a canonical hemodynamic function. Movement parameters computed during the realignment corrections (three translations and three rotations) were included in the design matrix of each session as additional parameters. Parameter estimates of activity in each voxel were generated using the general linear model at each voxel for each condition and each participant. Linear contrasts between the HRF estimates for the different experimental sessions were used to generate statistical parametric maps. All analyses were carried out with consistent and inconsistent trials separately as well as together. Results did not differ qualitatively between analyses; however all results presented here (behavioral and fMRI) were computed using consistent trials only.

At the individual level, statistical parametric maps were computed for several contrasts of interest. The entire cerebral network associated with ME processing was assessed by contrasting the ME condition to baseline (fixation point) conjointly for both character types (AN and nAN). The cerebral network associated with SE processing was assessed by contrasting the SE condition to baseline conjointly for both character types (AN and nAN). We identified brain regions involved more specifically in attention demanding simultaneous processing by contrasting the multiple to the SE condition for each character type. We then performed separate random-effect group analyses for control and dyslexic participants on the contrast images from individual analyses (Friston et al., 1998), using one-sample *t*-tests. Clusters of activated voxels were identified for each group, based on the intensity of the individual responses (Contrasts against baseline: voxel-wise threshold: *p* < 0.001 uncorrected for multiple comparisons, *T* > 4.0, with an cluster extent threshold correction of *p* < 0.05, Contrasts between conditions: voxel-wise threshold: *p* < 0.001 uncorrected for multiple comparisons, *T* > 4, with a cluster extent threshold of 20 voxels) Finally, two-sample *t*-tests were performed in order to statistically compare brain activity between controls and dyslexics on the relevant contrasts. Significance thresholds for between-group comparisons (voxel-wise threshold: *p* < 0.001 uncorrected for multiple comparisons, *T* > 3.5, with a cluster extent threshold of 20 voxels) were chosen by reference to previous studies reporting activation differences between skilled and dyslexic readers (Hoeft et al., 2007; van der Mark et al., 2009; Wimmer et al., 2010). For all analyses, brain regions were reported according to the Automated Anatomical Labelling SPM toolbox (Tzourio-Mazoyer et al., 2002).

#### *A priori* ROIs

Analysis was finally completed by statistically comparing activity for skilled and dyslexic readers within *a priori* anatomical ROIs. A first set of four ROIs was defined using predefined masks from the Wake Forest University (WFU) PickAtlas (Maldjian et al., 2003). ROI masks were created with the automated anatomical labeling atlas, which uses an anatomical parcellation of the MNI MRI single-subject brain and sulcal boundaries to define each anatomical volume. In order to assess neural activity in the part of the vOT cortex usually associated with character string processing, a second set of two *a priori* ROIs was defined by rectangular boxes. These ROIs were designed in reference to previous research (Jobard et al., 2003; Cai et al., 2010) within the bilateral fusiform and inferior temporal gyri rather than by anatomical boundaries. Parameter estimates (percent signal change) of event-related responses were then extracted from all ROIs for each participant. We both compared ROI activity between groups and tested whether activity levels in SPL covaried with activity levels in vOT. All ROIs were constructed using the SPM Marsbar toolbox (http://marsbar.sourceforge.net).

To investigate the presence of neural dysfunction in dyslexic participants, we first compared ROI activity between groups across different task conditions. To investigate putative links between neural activity in superior parietal cortex and in ventral occipital cortex for ME processing, we used multiple regression analyses to test whether percent signal change for the ME condition in SPL ROIs significantly predicted percent signal change in vOT ROIs while taking into account the putative effect of stimulus type. We ran separate regressions for each group (Dyslexic/Control) and hemisphere (Right/Left). The regression models tested were vOT ~ SPL + stimulus Type [stimulus Type was numerically coded as 0 (AN) or 1 (nAN)].

## RESULTS

### fMRI BEHAVIORAL RESULTS

Reaction times and accuracy for consistent trials during the fMRI task are presented in **Table 1**. For each condition, RTs and accuracy were entered in a 2 × 2 mixed design ANOVA with Group (Dyslexic vs. Control) as a between-subjects factor and character type (AN vs. nAN) as a within-subject factor. ME condition accuracy data were transformed in order to meet parametric assumptions. For the SE condition, there were no significant main effects or interaction (Group: *F*(1,22) = 4.1, *p* = 0.054, η^2^ = 0.11, Type: *F*(1,22) = 1.4, n.s., η^2^ = 0.02, Group × Type: *F*(1,22) = 0.08, n.s., η^2^ = 0.00). For ME RTs, the Type main effect was significant [*F*(1,22) = 7.5, *p* < 0.05, η^2^ = 0.05], as well as the Group × Type interaction [*F*(1,22) = 9.1, *p* < 0.01, η^2^ = 0.05] Type: [*F*(1,22) = 16.5, *p* < 0.001, η^2^ = 0.19]. The main effect of Group was not significant [*F*(1,22) = 2.9, n.s., η^2^ = 0.10]. Contrasts corrected for multiple comparisons showed that dyslexic participants are slower than control participants for AN character strings (*t*(22) = 2.8, *p* < 0.05) but not for nAN strings (*t*(22) = 0.5, n.s.). Accuracy for the SE condition was near ceiling for both groups. There were no significant main effects of Group [*F*(1,22) = 4.1, *p* = 0.053, η^2^ = 0.11] or Type [*F*(1,22) = 1.4, n.s., η^2^ = 0.02] and no significant Group × Type interaction [*F*(1,22) = 0.8, n.s., η^2^ = 0.00]. For accuracy in the ME condition, control participants were significantly more accurate than dyslexic participants [*F*(1,22) = 8.3, *p* < 0.01, η^2^ = 0.21], and participants were more accurate for AN strings than for nAN strings [*F*(1,22) = 16.5, *p* < 0.001, η^2 ^= 0.19]. The Group × Type interaction was not significant [*F*(1,22) = 2.0, n.s., η^2^ = 0.03], suggesting that the accuracy difference between dyslexic and control participants is the same regardless of character type.

**Table 1:** fMRI task performance of dyslexic and control participants for consistent trials.

	Dyslexics (*n* = 12)				Controls (*n* = 12)			
	Reaction time		Accuracy		Reaction time		Accuracy	
	Mean	95%CI	Mean	95%CI	Mean	95%CI	Mean	95%CI
Single element AN	772	689–857	0.95	0.89–1.0	690	637–743	0.99	0.98–0.1.0
Single element nAN	945	795–1095	0.95	0.92–0.99	812	729–894	0.98	0.96–1.0
Multiple element AN	1197	1040–1353	0.75	0.61–0.89	956	855–1057	0.94	0.90–0.97
Multiple element nAN	1187	1018–1356	0.66	0.58–0.73	1144	1030–1257	0.76	0.70–0.86

*
Reaction times are reported in ms, accuracy in proportion correct.*

### fMRI RESULTS

#### Within-group brain networks

**FIGURE 2:**
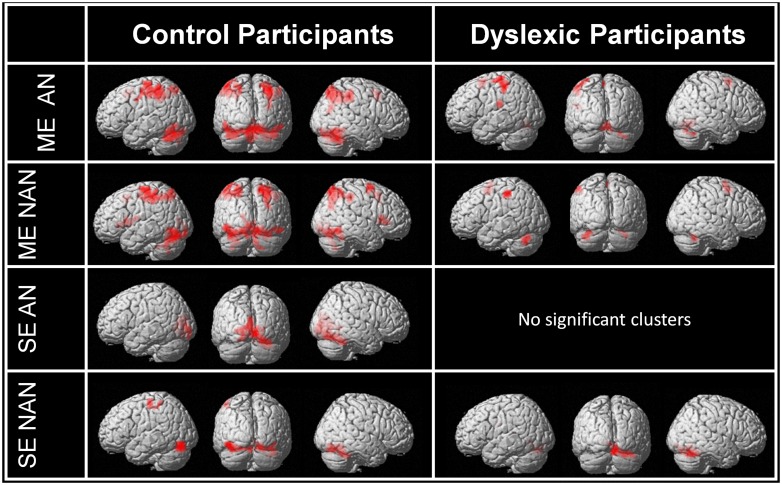
**Whole-brain activations induced by multiple and single element processing for AN and nAN conditions for control and dyslexic participants, overlaid on a surface-rendered single subject brain normalized to MNI template.** Top two rows: BOLD activation for the contrast [ME > Baseline] for each condition (AN and nAN) in control and dyslexic participants. Bottom two rows: BOLD activation evoked for the contrast [ME > Baseline] for each condition (AN and nAN) in control and dyslexic participants. For all contrasts: voxel-wise threshold of *p* < 0.001 uncorrected with an extent threshold correction of *p* < 0.05 at the cluster level.

First, we used contrasts between our task and baseline to identify the main networks of brain regions involved in multiple or SE processing in each group separately for AN and nAN character strings. Brain activations are illustrated in **Figure 2**. Relative to baseline (fixation) ME processing activated a broad and bilateral cortical network in control participants regardless of stimulus type. Visual areas included occipital extra-striate cortex bilaterally as well as fusiform and inferior temporal gyri bilaterally. Parietal activations extended over SPL and IPL bilaterally. Finally, cortical activations included the pre-supplementary motor area for AN characters as well as the right superior and middle frontal gyri for nAN characters. Dyslexic participants activated a more limited network. For AN characters; visual areas included the lingual gyrus. Parietal areas were limited to left IPL and postcentral gyrus. As with control participants, cortical activations included pre supplementary cortex. In addition, activation was present in the left rolandic operculum and supramarginal gyrus. The activation pattern was similar for nAN characters, save for the left rolandic operculum and supramarginal gyrus activity that was absent. Relative to baseline, SE processing activated a mostly ventral cortical network in control participants. For AN characters, a very limited network included the left calcarine, lingual gyrus, and cuneus as well as the right fusiform gyrus. For nAN characters; visual areas included occipital gyri and fusiform gyri bilaterally. Activated parietal areas were limited to the left postcentral and precentral gyri. For dyslexic participants, there were no significant activations at our chosen threshold for AN characters (Lowering the threshold revealed activation patterns similar to control participants). For nAN characters, activated visual areas included the right fusiform and bilateral lingual gyri.

**FIGURE 3:**
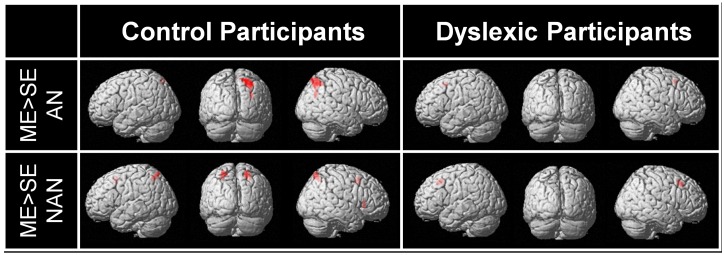
**BOLD activation for the contrast [ME > SE] for each condition (AN and nAN) and group (Control and Dyslexic), overlaid on a surface-rendered single subject brain normalized to MNI template.** For all contrasts: voxel-wise threshold of *p* < 0.001 uncorrected with a cluster threshold of 20 voxels.

For each group, brain regions specific to ME processing were identified by contrasting ME and SE conditions for each stimuli type (AN and nAN) separately. Brain areas showing stronger activations for the ME than the SE condition are listed in **Table 2** and illustrated in **Figure 3**. For control participants, the [*ME > SE*] contrast for AN strings activated a single right hemisphere parietal cluster. This cluster extended over parts of the superior and inferior parietal lobule as well as angular, superior occipital and mid occipital gyri. For nAN strings, control participants had stronger ME activations bilaterally in parietal cortex. A left hemisphere parietal cluster extended mainly over SPL (and over limited parts of precuneus and IPL) while the right hemisphere cluster extended exclusively over SPL. Increased activity was also found in the pre supplementary motor area. For dyslexic participants, the [*ME > SE*] contrast for AN and nAN characters revealed pre-supplementary motor area clusters in both conditions. Neither contrast revealed any parietal activation at the chosen threshold. No brain areas showed significantly stronger activity for the ME condition than for the SE condition in either group:..

**Table 2:** Cerebral regions significantly more activated for multiple element than for single element processing.

	Control group			Dyslexic group		
	*x, y, z*	*k*	*z*	*x, y, z*	*k*	*z*
[ME>SE] – AN	–	–	–	–	–	–
Parietal cortex	–	–	–	–	–	–
Right precuneus/superior parietal lobule	30, –60, 50	109	4.4	–	–	–
Bilateral pre-supplementary motor area	–	–	–	0, 12, 53	20	4.0
[ME>SE] – nAN	–	–	–	–	–	–
Parietal cortex	–	–	–	–	–	–
Right superior parietal lobule	21, –69, 56	24	3.6	–	–	–
Left superior parietal lobule/precuneus	–27, –60, 56	21	3.4	–	–	–
Insular cortex	–	–	–	–	–	–
Right insula/putamen	27, 24, 0	26	4.9	–	–	–
Bilateral pre supplementary motor area	12, 9, 49	34	3.9	6, 21, 46	26	3.9

*The statistical significance voxel-wise threshold of *p* < 0.001 uncorrected (*T* > 4.02) with an extent threshold correction of *p* < 0.05 at the cluster level. For each cluster, peak MNI coordinates (*x*,*y*,*z*), cluster spatial extent *k* and peak *z*-value are indicated. Anatomical labels are based on the automated anatomical labeling (AAL) atlas. Labels represent anatomical regions with the largest percentages of overlap with the activation cluster.*

#### Between-group differences in activation

**FIGURE 4:**
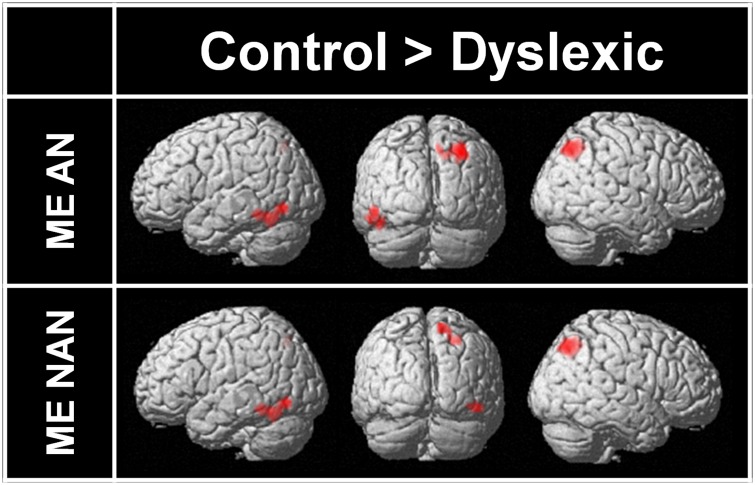
**Brain areas more strongly activated in control participants than in dyslexic participants for ME processing and AN or nAN characters, overlaid on a surface-rendered single subject brain normalized to MNI template.** For all contrasts: voxel-wise threshold of *p* < 0.001 uncorrected with a cluster threshold of 20 voxels.

Two-sample *t*-tests were then performed to statistically compare brain activation in control and dyslexic readers on relevant contrasts. To identify brain areas significantly more activated in normal readers than in dyslexic participants in ME processing, we compared activations for the ME condition between each group for each character type separately. Brain areas showing stronger activations for the control group than for the dyslexic group are listed in **Table 3** (ME and SE conditions) and illustrated in **Figure 4** (ME condition). For AN characters, the right parietal cortex (including SPL and extending to the superior part of the occipital cortex and precuneus) and the left vOT cortex (including the inferior temporal and fusiform gyri) were more strongly activated in control than dyslexic readers. For nAN characters, there were stronger activations for control than dyslexic participants in the right parietal cortex (including SPL and precuneus) as well as in the right vOT cortex (including inferior temporal and inferior occipital gyri). The opposite comparison ([Dyslexic > Control]) revealed no areas more activated for dyslexic than for control participants for either character type.

**Table 3:** Overview of clusters significantly more activated for one group compared to the other [control > dyslexic and control > dyslexic; voxel-wise threshold of *p* < 0.001 uncorrected (*T* > 3.5) with a cluster extent *k* > 20].

	Control > Dyslexic				
	*x, y, z*	*k*	*z*
[ME - AN > Baseline]			
Parietal cortex			
Right superior parietal lobule/superior occipital gyrus	33, -69, 46	100	4.4
Temporo-occipital cortex			
Left inferior temporal/fusiform gyri	-45, -57, -21	59	4.2
[ME - nAN > Baseline]			
Parietal cortex			
Right superior parietal lobule/precuneus	15, -72, 63	23	3.5
Temporo-occipital cortex			
Right inferior temporal/inferior occipital gyri	48, -63, -11	23	3.8
	
	**Dyslexic > Control**				
[SE- AN > Baseline]			
Frontal cortex			
Left frontal middle/superior gyri	-24, 24, 32	23	4.4

*For each cluster, peak MNI coordinates (*x*,*y*,*z*), cluster spatial extent *k* and peak *z*-value are indicated. Anatomical labels are based on the AAL [(automated anatomical labeling) atlas (Tzourio-Mazoyer et al., 2002)]. Labels represent anatomical regions with the largest percentages of overlap with the activation cluster. Contrasts with no significant clusters are not presented.*

We then compared activations for SE processing between each group by contrasting activations maps ([Control > Dyslexic]) for the SE condition separately for each character type (AN and nAN) There were no brain areas significantly more activated in control than in dyslexic participants for either character type. The opposite contrasts ([Dyslexic > Control]) showed that for AN characters, a single left middle/superior frontal gyri cluster was more strongly activated in dyslexic than control participants (see **Table 3**). For nAN characters, there were no brain areas significantly more activated in dyslexic than in control participants.

#### Regions of interest

**FIGURE 5:**
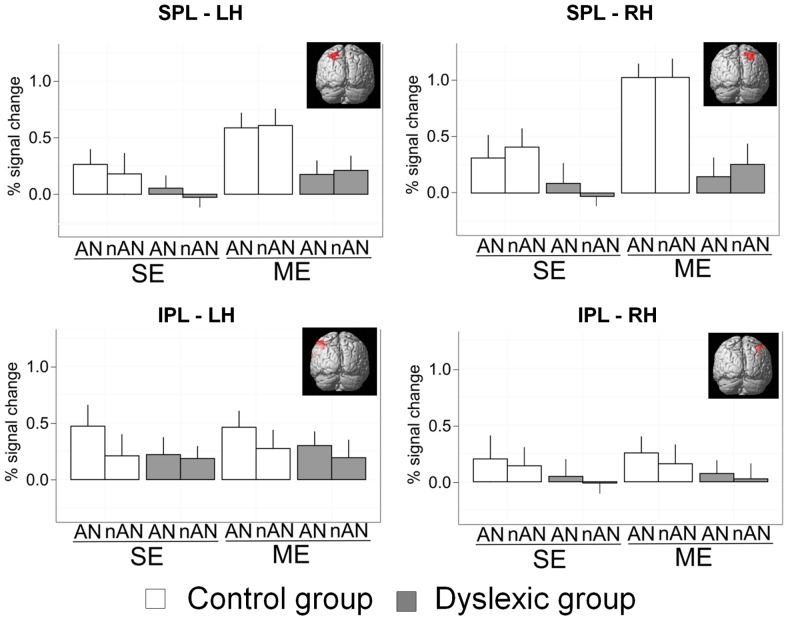
**Mean percent signal change for *a priori* SPL and IPL ROIs.** Error bars indicate standard error.

Previous research has linked behavioral deficits in simultaneous visual processing in dyslexia to lower activation in parietal brain areas, and more specifically in the SPL bilaterally and the left inferior parietal lobule (Peyrin et al., 2011; Reilhac et al., 2013). We compared parietal activations in dyslexic and skilled readers in four predefined and standardized neuro-anatomical ROIs using predefined masks from the WFU PickAtlas (Maldjian et al., 2003). The first two ROIs were defined as right and left SPL intersected with BA7 and the next two as right and left IPL intersected with BA 40 (as defined by the automated labeling atlas which uses an anatomical parcellation of the MNI single subject brain and sulcal boundaries to define anatomical volumes). The SPL/BA7 ROI sizes were, respectively, of 139 (R) and 136 (L) voxels. The IPL/BA40 ROI sizes were, respectively, of 333 (R) and 367 (L) voxels (ROIs are illustrated in **Figure 5**). Parameter estimates (percent signal change) were extracted for each ROI and entered in a 2 × 2 × 2 mixed ANOVA with Condition (ME vs. SE) and Character Type (AN vs. nAN) as within-subject factors as well as Group (Dyslexic vs. Control) as a between-subject factor (see **Figure 5**). Concerning right SPL, there were significant main effects of Condition [*F*(1,22) = 21.3, *p* < 0.0001, η^2^ = 0.13] and Group [*F*(1,22) = 12.5, *p* < 0.01, η^2^ = 0.22] as well as a significant Group × Condition interaction [*F*(1,22) = 7.5, *p* < 0.05, η^2^ = 0.05]. There was neither a significant main effect of character type nor any other significant interaction. The difference in activation between groups was affected by the number of elements to be processed. Contrasts indicated that the interaction was driven by a different effect of Group in each Condition. The effect of Group was significant for the ME condition [*F*(1,22) = 20.4, *p* < 0.001], but non-significant in the SE condition [*F*(1,22) = 3, n.s.]. Concerning left SPL, there were significant main effects of Condition [*F*(1,22) = 11.9, *p* < 0.01, η^2^ = 0.09] and Group [*F*(1,22) = 8.4, *p* < 0.05, η^2^ = 0.11]. No other effects were significant. The difference in activity between groups in left SPL is not affected by condition demands. Concerning IPL, results were similar for right and left hemisphere. There were no significant main effects for either Group [RH: *F*(1,22) = 1.1, n.s.; LH: *F*(1,22) = 0.7, n.s.], Condition [RH: *F*(1,22) = 0.1, n.s.; LH: *F*(1,22) = 0.1, n.s.] or Character Type [RH: *F*(1,22) = 0.6, n.s.; LH: *F*(1,22) = 3.2, n.s.], suggesting that IPL is not specifically implicated in ME processing in either healthy or dyslexic readers.

**FIGURE 6:**
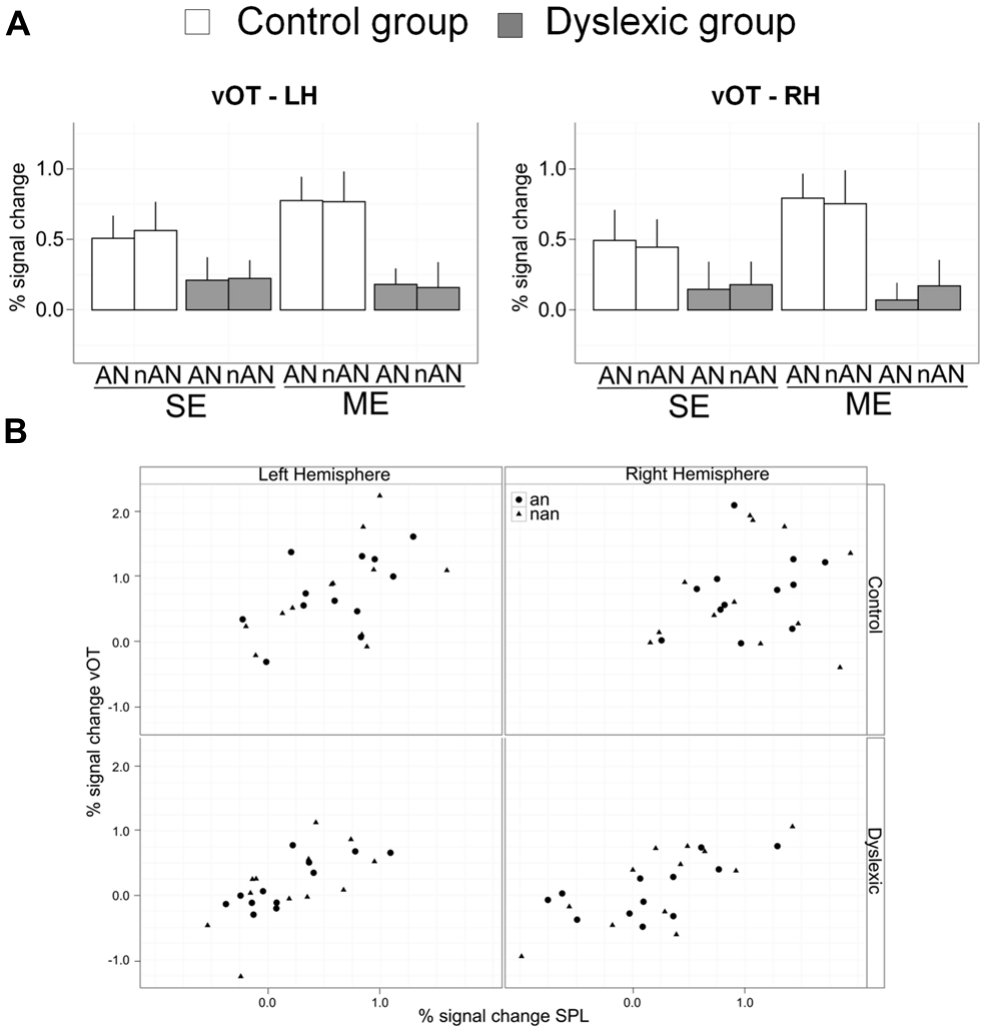
**(A)** Mean percent signal change for *a priori* vOT ROIs. Error bars indicate standard error. **(B)** Scatterplots of vOT mean percent signal change as a function of SPL mean percent signal change for multiple element processing. Each combination of Hemisphere (Left/Right) and Group (Control/Dyslexic) is represented.

Abnormal brain activity for letter strings in the left vOT cortex in dyslexia is well documented (see Richlan et al., 2011 for a recent meta-analysis). We built a ROI covering the fusiform and inferior temporal gyri using a coordinate-delimited box (RH: *X* = –34 to –55, *Y* = –34 to –68, *Z* = –4 to –26, mirror-reversed for LH). This ROI was defined by Cai et al. (2010) according to activation peaks reported in meta-analysis of normal word reading by Jobard et al. (2003). Parameter estimates were extracted and analyzed similar to SPL and IPL ROIs (See **Figure 6A**). In the right hemisphere ROI, there was a significant main effect of Group [*F*(1,22) = 7.5, *p* < 0.05, η^2^ = 0.13] and no other effects were significant [Condition: *F*(1,22) = 1.5, n.s., η^2^ = 0.01; Type: *F*(1,22) = 0.01, n.s., η^2^ = 0.00]. The result pattern was similar in the left hemisphere with a significant main effect of Group [*F*(1,22) = 7.5, *p* < 0.05, η^2^ = 0.14] and no other significant effects [Condition: *F*(1,22) = 1.3, n.s., η^2^ = 0.01; Type: *F*(1,22) = 0.01, n.s., η^2^ = 0.00]. Reduced brain activity in the vOT cortex for dyslexic participants is present for single or ME processing as well as for AN or nAN character strings.

To investigate putative links between neural activity in superior parietal cortex and in ventral occipital cortex, we ran regressions for each group and hemisphere with percent signal change in vOT ROIs as the dependent variable and percent signal change in SPL ROIs as well as stimulus type as regressors (Scatterplots of the data are shown in **Figure 6B**). The effect of stimulus type was non-significant in all regressions, suggesting that a putative link between vOT and SPL is independent of character type. In the right hemisphere, SPL predicted vOT for the dyslexic group [Full regression: *F*(2,21) = 10.1, *R*^2^ = 0.49, *p* < 0.001, SPL regressor: β = 0.6, *t* = 4.5, *p* < 0.0001], but, not for the control group [Full regression: *F*(2,21) = 0.5, *R*^2^ = 0.05, n.s., SPL regressor: β = 0.3, *t* = 1.0, n.s.]. In the left hemisphere, SPL predicted vOT for the dyslexic group [Full regression: *F*(2,21) = 8.9, *R*^2^ = 0.46, *p* < 0.01, SPL regressor: β = 0.8, *t* = 4.2, *p* < 0.0001], as well as for the control group [Full regression: *F*(2,21) = 4.3, *R*^2^ = 0.29, *p* < 0.05, SPL regressor: β = 0.6, *t* = 3.0, *p* < 0.01].

## DISCUSSION

The present fMRI study compared character string processing in VA Span impaired dyslexic readers and healthy skilled readers. Reduced performance of dyslexic participants on a 6-letter global report compared to control participants is posited to index a general impairment of parallel ME processing. This VA Span impairment has been associated with reduced SPL activation for multiple letter processing in dyslexic children (Peyrin et al., 2011). The main purpose of this study was to extend these results to nAN character processing. We hypothesized that abnormal parietal activations should be found in dyslexic individuals with a VA span disorder regardless of character type for ME processing. In addition, we hypothesized that if parietal cortex is involved in visual processing and information extraction from multiple character strings, then parietal activity should correlate with vOT activity for character string processing. Participants carried out a visual categorization task in two conditions: SE or MEs. The task was carried out with alphanumeric, familiar characters and non-alphanumeric, unfamiliar characters in order to investigate the stimulus specificity of the putative parallel ME processing deficit.

Dyslexic participants for this study were selected to present a VA Span deficit at the individual level. VA Span abilities were assessed outside the scanner, using a 6-letter whole report paradigm similar to the 5-letter paradigm used with children (Bosse et al., 2007; Bosse and Valdois, 2009). Dyslexic participants were not able to report as many letters from a briefly presented array of letters as normal-reading adults. This behavioral impairment is taken as indexing a reduced ability to attend to and process MEs simultaneously. Dubois et al. (2010) showed that a reduced VA Span co-occurred with reduced VA capacity for MEs in dyslexic children while Stenneken et al. (2011) provide similar evidence for reduced VA capacity in high achieving dyslexic adults. In our experimental fMRI task, dyslexic participants were expected to perform as well as control participants for the SE condition, but to perform significantly worse for the ME condition, in line with a specific ME processing deficit. Furthermore, the ME processing behavioral impairment has been associated with abnormal brain activations in the parietal cortex, and more specifically in SPL. Comparisons between activations for ME processing in control and dyslexic participants were expected to highlight abnormal parietal neural activity in dyslexia, regardless of to-be-processed character type.

Behavioral results are consistent with a specific ME processing deficit regardless of character type. Both groups performed at ceiling for SE categorization, although RTs were slower for nAN characters than for AN characters for both groups. For the ME condition, dyslexic participants were less accurate than control participants regardless of character type, but were slower only for AN characters. Reduced accuracy for both character types argues for a general inability to attend to and process all displayed elements in VA Span impaired dyslexics. The different pattern of results for RTs could be explained by accuracy and RTs indexing different processes in character recognition for short exposure durations (Santee and Egeth, 1982). While accuracy could be sensitive to early perceptual effects, RTs could be more sensitive to later processes such as response interference. Within such a framework, poor VA capacity (an early process) would lead to poorer accuracy for dyslexic participants regardless of character type. Interference by later processes could be stronger when the task is not performed at ceiling performance levels, resulting in slowed RTs for dyslexic participants for both character types and in slowed RTs for control participants only for nAN characters.

### NEURAL CORRELATES OF SINGLE AND MULTIPLE ELEMENT PROCESSING IN HEALTHY, SKILLED READERS

In control participants, ME processing recruited additional regions from a broad occipito-parietal network compared to SE processing (see **Figure 2**). ME processing activated vOT cortex, as expected for processing single (Flowers et al., 2004) or multiple letters and symbols (Tagamets et al., 2000; Turkeltaub et al., 2003; Brem et al., 2006). However, patterns of parietal activation differed. In SE processing, there were no significant parietal activations. In contrast, ME processing activated a broad parietal network, including SPL, IPL, and precuneus bilaterally. Involvement of IPL and SPL in VA processes is well documented (Behrmann et al., 2004), and could be related to the attentional demands of attending to several characters. Furthermore, activations of SPL and IPL for multiple character processing are consistent with reports of similar activations in adult healthy skilled readers for letter string processing (Levy et al., 2008; Valdois et al., 2009), a flanked character categorization task (Peyrin et al., 2008) or a visual matching task (Reilhac et al., 2013), and in typically reading children for the same flanked character categorization task (Peyrin et al., 2011).

Brain areas specifically involved in ME processing in healthy readers were identified by contrasting ME to SE conditions for each stimuli type (AN and nAN) separately. ME processing activated parietal cortex more strongly than SE processing for both character types. For nAN characters, additional increased activation were located in the right insula, as have been previously reported in VA tasks (Hahn et al., 2006), and in the pre supplementary motor area consistent with that area’s putative role in cognitive processes (Picard and Strick, 2001). Increased SPL activity for ME processing was limited to the right hemisphere for AN characters while bilateral for nAN characters. Similar recruitment of left-side homologues for VA tasks with high cognitive demands has been previously reported (Nebel et al., 2005). SPL activations are broadly consistent with our team’s previous studies investigating neural correlates of ME processing (Peyrin et al., 2008, 2011), albeit specific activity seems to be more right lateralized in this study. As parietal activity has consistently been associated with visuo-spatial attention (Corbetta and Shulman, 2002; Behrmann et al., 2004), increased parietal activations for both conditions (AN and nAN) could index increased demands on VA for the processing of MEs.

### NEURAL CORRELATES OF SINGLE AND MULTIPLE ELEMENT PROCESSING IN DYSLEXIC READERS

Neural networks associated with single and ME processing were more limited in dyslexic participants. For SE processing, visual processing activity was limited to the occipital and occipito-temporal cortices. ME processing in dyslexic readers failed to elicit the broad parietal network present for control participants. Although similar pre-supplementary motor area activations were present for both groups, parietal activations for dyslexics were limited to the left supramarginal gyrus and post-central gyrus. This relative absence of parietal activation is consistent with previous assessments of neural activity for multiple letter processing in dyslexic participants with poor VA Span performance (Peyrin et al., 2008, 2011, 2012; Reilhac et al., 2013; Valdois et al., 2014b).

Further assessment of neural networks subserving ME processing was carried out by contrasting multiple and SE processing for each character type. Similarly to control participants, ME processing led to increased pre-supplementary motor area activations in both conditions (AN and nAN). This pre-supplementary motor area activity, present for more demanding task conditions (ME > SE AN and nAN for dyslexic participants, but also ME > SE nAN for control participants) could reflect higher cognitive demands (Picard and Strick, 2001). However, a complete absence of parietal activation in either hemisphere, for either character type, is to be noted. This absence of parietal activations could reflect a failure to engage appropriate attentional mechanisms for processing MEs, failure that would then lead to impaired behavioral performance.

### MODULATION OF MULTIPLE ELEMENT PARIETAL ACTIVATIONS BY READING ABILITY

To identify brain areas significantly more activated in normal readers than in dyslexic participants in ME processing, we compared activations for the ME condition between each group for each character type separately. For both character types (AN and nAN), control participants had larger activations in broadly similar areas in both ventral and dorsal cortices. Reduced activity in vOT cortex was present in the left hemisphere for AN characters and in the right hemisphere for nAN characters. Consistent with the difference in ME processing activity patterns between groups, dyslexic participants exhibit reduced activation in right hemisphere SPL regardless of character type. While previous studies have hinted towards a left SPL dysfunction in VA Span impaired dyslexics (Peyrin et al., 2008, 2011), the current findings seem to point to right SPL as the critical area subserving successful ME processing.

Taken together, results from these whole-brain analyses point towards a right hemisphere superior lobule dysfunction in VA Span impaired dyslexic adults. This functional impairment of parietal cortex seems to be condition-related (present in multiple but not in SE processing) but not stimuli-type related (equally large for NA and nAN characters). Furthermore, this pattern of dysfunction is localized to SPL. This account is supported by our *a priori* ROI analyses. For right hemisphere SPL, the difference in activation between groups was affected by the number of elements to be processed (the activation difference was present for ME processing but absent for SE processing). Interestingly, although whole-brain comparisons between groups did not reveal any left hemisphere activation differences, ROI analyses of left SPL showed stronger activations for normal readers for both ME and SE processing.

A possible confounding factor in these results is the difference in behavioral performance between groups. Differences in neuronal activity could reflect lower accuracy for dyslexic participants within a functional parietal network rather than a dyslexic parietal dysfunction. It, however, seems unlikely that between-group differences in neuronal activation only resulted from between-group differences in RTs, since between-group neuronal activity differences were present for the ME-nAN condition in the absence of between-group RTs differences.

The critical result of this study is that this parietal dysfunction is present regardless of character type. Whole-brain comparisons between groups for the ME-nAN condition revealed dyslexic under-activation in right hemisphere SPL clusters. Indeed, result patterns in SPL ROIs suggested that activations did not differ between character types, and this was true for both dyslexic and control participants. The activation difference between control and dyslexic participants is the same for AN, familiar, verbal characters, and nAN, unfamiliar, non-verbal characters. This strongly suggests the existence of abnormal neural function in dyslexia in non-language related processes.

Finally, this pattern of condition sensitive/stimuli non-sensitive deficit seems to be circumscribed to right SPL. Activation patterns in other parietal (left SPL, bilateral IPL) or upper visual areas (bilateral vOT) were explored in our *a priori* ROI analyses. Bilateral IPL is equally activated for ME or SE conditions, suggesting it plays no specific role in ME processing. This is supported by the absence of activation strength differences between dyslexic and control participants for either the ME or SE conditions. There were also stronger activations for control participants than dyslexic participants in vOT and left SPL. However, this activation difference between groups was similar for (1) SE and ME conditions and (2) for AN and nAN character strings. Within the constraints of our experimental paradigm, VOT BOLD activity seems to be sensitive to neither VA demands nor character type.

### IMPLICATIONS FOR THE VA SPAN HYPOTHESIS OF DYSLEXIA

While previous studies had reported decreased activations in SPL for ME processing in VA Span impaired dyslexia (Peyrin et al., 2011, 2012; Reilhac et al., 2013; Valdois et al., 2014b), this is the first study to do so by using a non-verbal task requiring verbal and non-verbal stimuli processing. Our results bring forward new evidence for a visual-attention account of the VA Span deficit. Indeed, these data speaks against two alternative explanations of poor dyslexic performance on the VA Span letter report tasks: impaired print tuning and impaired object-to-phonological code mapping. While our results do not rule out impaired print tuning as one of the contributing factors to poor letter report performance, they argue against it being the sole cause. If poor letter report performance only indexed reduced perceptual specialization for letter (Nazir et al., 2004) or letter-like character (Szwed et al., 2012) strings in dyslexia (Maurer et al., 2007; van der Mark et al., 2009), we would expect poor performance on our ME categorization task to be associated with activation differences in visual rather than parietal cortex. If poor letter report performance were a consequence of impaired visual-to-phonological code mapping (Hawelka and Wimmer, 2008; Ziegler et al., 2010 but see Valdois et al., 2012), we would expect dyslexic participants to perform as well as control participants on a non-verbal categorization task, even more so for non-verbal stimuli. In contrast and in line with similar behavioral results previously reported with typical reading children (Lobier et al., 2012b), dyslexic participants performed worse than control participants in the ME condition. Furthermore, impaired visual-to-phonological code mapping would not result in abnormal brain activity for dyslexic individuals for visual processing of non-verbal character strings, as is present in our data. In contrast, decreased activation of right hemisphere SPL, a brain area consistently associated with space-based (Vandenberghe et al., 2001; Yantis et al., 2002) and object-based (Yantis and Serences, 2003) attention, could index impaired ability to properly attend to MEs simultaneously. SPL could subserve two necessary attentional mechanisms: chunking character strings into appropriate individual elements and allocating spatial attention to each individual element to allow further processing. This could be done by modulating lower level visual responses to spatial locations or features (Corbetta and Shulman, 2002). If all visual elements cannot be attended to in our ME categorization condition, target characters may be missed, leading to poor performance. Similarly, if dyslexic participants can attend to fewer letters than control participants in the VA Span letter report task, their performance will be worse. Poor performance or neurobiological dysfunction cannot be ascribed to different amounts of lifelong experience with characters between dyslexic and control participants. First, all participants had the same amount of limited experience with the nAN characters. Second, SPL parietal dysfunction is of similar magnitude regardless of stimuli type, consistent with similar parietal activation patterns for letter and non-letter stimuli (Nebel et al., 2005). In sum, abnormal parietal activations in VA Span impaired dyslexic participants for ME processing of both AN and nAN character strings supports a ME visual processing disorder as the underlying cause of the VA Span deficit.

### IMPLICATIONS FOR NEUROBIOLOGICAL MODELS OF DYSLEXIA

Neurobiological accounts of dyslexia, in line with classic models of reading usually highlight neural dysfunction of the left hemisphere reading network as a hallmark of dyslexia. These functional deficits are present in brain areas thought to subtend phonological processing (left inferior frontal, and parieto-temporal gyri) and orthographic word processing (vOT cortex; see Shaywitz and Shaywitz, 2005 for a review). These abnormal brain activations are identified using reading or reading related tasks (e.g., rhyming) and verbal visual stimuli, in line with a phonological account of dyslexia. The overwhelming developmental model of this disruption of reading neural circuits is one where the vOT neural dysfunction systematically follows from frontal and temporo-parietal dysfunction (McCandliss and Noble, 2003): impaired phonological processing impedes the acquisition of orthographic knowledge and the development of appropriate neural tuning for print (Maurer et al., 2007; van der Mark et al., 2009). However, this model fails to account for a number of empirical findings. First, there is mounting evidence that while a number of dyslexic children do in fact have a phonological deficit, a non-negligible number do not (White et al., 2006; Bosse et al., 2007; Vidyasagar and Pammer, 2010). In line with these behavioral results, a recent case study has reported not only normal phonological behavioral performance but also normal activation of the fronto-temporo-parietal network associated with phonological processing (Peyrin et al., 2012). Second, a recent meta-analyses of brain imaging studies of dyslexic children and adults has failed to find unilateral evidence for a contrasted pattern of predominant left temporo-parietal dysfunction in children and predominant left vOT dysfunction in adults (Richlan et al., 2011). These results suggest that reduced print tuning and orthographic specificity of left vOT cortex in dyslexia could follow from alternative disruption in the learning to read process.

Two aspects of our data are noteworthy. As expected from our hypotheses and appropriately highlighted earlier, VA Span impaired dyslexic adults display reduced parietal activations in tasks requiring visual processing of multiple characters, AN or not. More unexpectedly, task related activations were also reduced in vOT cortex bilaterally and for both character types. Previous accounts of reduced vOT in dyslexia have been associated with processing of letter strings (word or non-words) and restricted to LH vOT (Helenius et al., 1999; Maurer et al., 2007; van der Mark et al., 2009; Wimmer et al., 2010). Indeed, neural responses for non-alphabetic strings have usually been similar in dyslexic and control readers (Helenius et al., 1999; van der Mark et al., 2009 but see Maurer et al., 2007). However, an important caveat of these studies is that their experimental tasks required no explicit processing of individual elements of the non-alphabetic strings. In contrast, in our study, explicit processing of the individual characters composing strings is necessary for both character type. Therefore, if visual processing of individual elements in vOT is influenced by top-down VA related parietal activity, then a parietal dysfunction should result is abnormal vOT activity regardless of character type. In addition, while the difference in vOT activity between letter and non-letter string processing is present only in left vOT in expert readers, visual processing of both string types recruits vOT bilaterally (Tagamets et al., 2000; Vinckier et al., 2007). If at least part of this vOT activity is top-down driven by parietal cortex, then abnormal parietal function will result in abnormal vOT activity bilaterally. The presence of consistent correlations between SPL and vOT activity in each hemisphere further argues for this interpretation of our data. We posit that not only these two co-occuring neural dysfunctions (SPL and VOT) are related but that this relationship can explain disrupted vOT function in dyslexic readers independently from any phonological deficit.

How can impaired parietal function lead to decreased vOT activity in a ME processing task? Parietal areas are responsible for feature and spatial attention focus and shifts (Kanwisher and Wojciulik, 2000). Dorsal areas are thus involved in a fast feedforward/feedback loop with visual areas: early visual signals trigger parietal attention mechanisms and global analysis which then guides further processing in the ventral stream (Bullier, 2001). If attentional processes fail, the downstream ventral processing is also disrupted. In our task, failure to allocate attention appropriately to each element of the character string reduces feedback to ventral areas responsible for character recognition (Szwed et al., 2011) and thus leads to reduced occipito-temporal activations. How does this relate to impaired vOT specificity for print in dyslexia? When children learn to read, they cannot at first rely on fast, parallel processing of words as supported by vOT in expert readers (Dehaene and Cohen, 2011). Letter string processing is supported by attention-based processes as supported by parietal cortex. Development of orthographic knowledge in vOT is therefore dependent on appropriate attentional feedback from parietal areas for proper letter identification. Similar involvement of parietal areas in reading is seen when spatial layout of words is modified in order to disrupt automatic vOT processing (Mayall et al., 2001; Pammer et al., 2006; Cohen et al., 2008; Rosazza et al., 2009). If parietal function fails, vOT specialization cannot take place and fast, automatic visual word processing cannot be achieved. In line with such a model, Richlan (2012) has proposed that impaired general attention processes in dyslexic readers, indexed by abnormal left IPL activity, could result in lack of vOT specialization for print.

Recent connectivity studies in normal and dyslexic readers offer support for this account. Both resting-state and functional connectivity between parietal areas and vOT have been reported, and this connectivity is modulated by reading efficiency. Vogel et al. (2011) investigated resting state connectivity between the specific part of vOT cortex thought to subserve orthographic reading, namely the visual word form area (VWFA; Cohen and Dehaene, 2004; Dehaene and Cohen, 2011) and the dorsal attentional network. They not only found significant connectivity between the VWFA and superior parietal cortex bilaterally, but this connectivity was significantly correlated to reading ability. Better readers had stronger connectivity between SPL and VWFA. Van der mark et al., 2011 investigated functional connectivity between five different seed regions of left vOT cortex (including the VWFA) and other brain regions in normal-reading and dyslexic children. In normal-reading children, bilateral SPL was significantly correlated to the middle, VWFA proper, seed area. This correlation between bilateral SPL and the VWFA seed area did not reach significance in dyslexic children (In that study, however, that the difference in functional connectivity between normal reading and dyslexic children did not reach significance for SPL-VWFA but did for left IPL-VWFA). Taken together, these results speak strongly for an important role of SPL in efficient reading.

In line with the VA span hypothesis of dyslexia (Bosse et al., 2007), VA Span impaired dyslexic adults are impaired in a non-verbal ME processing task. This impairment is associated with reduced specificity of SPL for ME processing, in support of a visual account of the VA span deficit. Co-occurring reduced vOT activation could be related to reduced connectivity between dorsal and ventral visual areas, in line with recent accounts of reduced SPL-vOT connectivity in dyslexia. Further research is needed to (1) investigate if and how the time-course of parietal and vOT activity in ME processing tasks deviates in dyslexic participants and (2) assess connectivity between SPL and vOT in both normal-reading and dyslexic readers with a VA span disorder.

## Conflict of Interest Statement

The authors declare that the research was conducted in the absence of any commercial or financial relationships that could be construed as a potential conflict of interest.
